# Who are the adult women exposed to violence in Brazil?

**DOI:** 10.11606/s1518-8787.2025059005701

**Published:** 2025-04-07

**Authors:** Nádia Machado de Vasconcelos, Crizian Saar Gomes, Juliana Bottoni de Souza, Fabiana Martins Dias de Andrade, Regina Tomie Ivata Bernal, Elaine Leandro Machado, Adalgisa Peixoto Ribeiro, Deborah Carvalho Malta

**Affiliations:** I Universidade Federal de Minas Gerais. Faculdade de Medicina. Programa de Pós-Graduação em Saúde Pública. Belo Horizonte, MG, Brasil; II Universidade Federal de Minas Gerais. Escola de Enfermagem. Observatório de Doenças e Agravos Não Transmissíveis. Belo Horizonte, MG, Brasil; III Universidade Federal de Minas Gerais. Escola de Enfermagem. Programa de Pós-Graduação em Enfermagem. Belo Horizonte, MG, Brasil; IV Universidade Federal de Minas Gerais. Faculdade de Medicina. Departamento de Medicina Preventiva e Social. Belo Horizonte, MG, Brasil; V Universidade Federal de Minas Gerais. Escola de Enfermagem. Departamento de Enfermagem Materno Infantil e Saúde Pública. Belo Horizonte, MG, Brasil

**Keywords:** Gender-Based Violence, Violence Against Women, Cross-Sectional Studies, Brazil

## Abstract

To estimate the prevalence of violence subtypes and analyze some demographic, socioeconomic, and health factors associated with violence against women in Brazil.

Cross-sectional epidemiological study using the 2019 National Survey of Health. The prevalences of some violence subtypes (psychological, physical, and sexual) in the 12 months prior to the interview were estimated in the country, per federative units. The characteristics of each subtype of violence were also analyzed. Additionally, the crude and adjusted prevalence ratios were estimated by a multivariate model according to the following potential demographic, socioeconomic, and health associated factors: age group, education, skin color, place of residence, household income, marital status, social support network, self-rated health, alcohol consumption, depression, and sexually transmitted infections.

In 2019, 19.38% of Brazilian women reported experiencing violence, with psychological violence being the most common subtype both in isolation and in conjunction with other subtypes. The main aggressor was an intimate partner and most of the violent acts occurred at home, with more than half of women reporting at least one consequence of these acts. Younger women, those with worse self-rated health, alcohol consumption, depression, and sexually transmitted infections had a higher prevalence of all violence subtypes.

One in five Brazilian women reported experiencing violence in the past 12 months. Violence against women is positively associated with younger ages, lower education, Black and Brown skin color, smaller support networks, and health-related factors such as self-rated health, alcohol consumption, depression, and sexually transmitted infections.

## INTRODUCTION

Violence Against Women (VAW), defined as “any act or conduct of gender-based violence that results in death, harm, or physical, sexual, or psychological suffering to women, whether occurring in public or private life”^1^, constitutes a social and public health problem. It is a multicausal phenomenon associated with economic inequalities and subjective and behavioral aspects prevalent in society^2^.

Globally, one in three women over the age of 15 has experienced physical and/or sexual violence during her life, with a similar prevalence in the Americas^3^. In Brazil, 35.60% of female homicides recorded in 2022 were classified as femicides, the lethal form of VAW^4^.

Women exposed to VAW are more likely to develop mental health disorders, engage in risky behaviors, and contract sexually transmitted infections (STIs)^1^. Furthermore, VAW significantly impacts the national economy, with a previous study showing that, over a decade, this type of violence reduced Brazil’s Gross Domestic Product by R$ 214.42 billion^5^.

VAW is linked to social inequalities, and low levels of education and economic status can influence its occurrence^2^. However, women’s difficulty recognizing violence and the lack of adequate training for healthcare professionals to identify indirect signs of this condition lead to underreporting of VAW, complicating data collection and limiting understanding of the reality of this problem in the country^6^.

There is a lack of nationwide studies in Brazil that have estimated VAW, its subtypes, and potential associated factors. In this sense, this study aimed to estimate the prevalence of subtypes of violence and analyze some of the demographic, socioeconomic, and health factors associated with VAW in Brazil.

Understanding the complex interaction of VAW and other population-level factors at a national level is essential for planning and implementing Public Policies focused on women in situations of violence, with particular attention to those in social vulnerability.

## METHODS

### Design and Data Source

This is a cross-sectional, analytical epidemiological study using data from the National Survey of Health (PNS) conducted in 2019.

The PNS is a household survey that uses a representative sample of the Brazilian population, reflecting distribution by sex, age group, skin color, household income, and macro-region of residence, among others. The 2019 PNS sample on a three-stage cluster sampling design: : i) census sectors or groups of sectors, ii) households, and iii) residents. A total of 90,846 interviews were conducted, which corresponded to a 96.50% response rate. For this study, women aged 18 or over from across Brazil who responded to the violence module (n = 46,869) were selected. Details of the 2019 PNS methodology are available in a previous publication^7^.

### Variables

The 2019 PNS questionnaire investigated exposure to three subtypes of violence, asking whether, in the past 12 months, someone:

1. Offended, humiliated, or ridiculed you in front of others?; yelled at you or swore at you?; used social media or a cell phone to threaten, offend, swear at, or expose images of you without your consent?; verbally threatened to hurt you or someone important to you?; destroyed something of yours on purpose? (psychological violence)
2. Slapped or smacked you?; pushed you, held you tightly, or threw something at you to hurt you?; punched, kicked, or dragged you by the hair?; attempted or actually strangled, suffocated, or burned you on purpose?; threatened or injured you with a knife, firearm, or any other weapon or object? (physical violence)
3. Touched, manipulated, kissed, or exposed parts of your body against your will?; threatened or forced you to have sexual relations or any other sexual acts against your will? (sexual violence)

This study considered exposure to a particular subtype of violence if a woman reported experiencing at least one of the situations outlined in that subtype. Additionally, the variable “Any Violence” was developed, which includes the indication of an affirmative response to at least one of the subtypes of violence.

Among the women who reported violence, the following variables derived from the questions by subtype of violence were analyzed:

- Aggressor: intimate partner, family member, acquaintance, and others
- Location: residence, work/study location, public place, and others
- Recurrence: yes or no
- Health consequences: a) psychological: fear, sadness, discouragement, difficulty sleeping, anxiety, depression, or other psychological consequences; b) physical: bruises, cuts, fractures, burns, or other physical injuries or wounds; and c) sexual: sexually transmitted disease or unwanted pregnancy.

For women who reported health consequences from violence, healthcare-seeking and the need for hospitalization for more than 24 hours were analyzed.

To assess the potential factors associated with VAW, some explanatory variables related to the demographic, socioeconomic, and health characteristics of women were selected:

a. Demographic:

- Age range: 18 to 24 years, 25 to 39 years, 40 to 59 years, and 60 years or older.
- Education: no education and incomplete primary education; complete primary school and incomplete secondary education; complete secondary education and incomplete higher education; and higher education.
- Skin color: White, Brown/Mixed-race, or Black.
- Place of residence: urban or rural.

b. Socioeconomic:

- Household income: up to 1 minimum wage (MW); more than 1 to 3 MW; above 3 MW.
- Marital status: single; married; widowed; divorced, separated, or legally separated.
- Social support network: number of people the woman could rely on in good or bad times (none; one; two; three or more);

c. Health:

- Self-rated health: very good or good; fair; poor or very poor;
- Alcohol consumption: consumption of 8 or more doses of alcohol per week (yes or no);
- Depression: diagnosis of depression by a physician or mental health professional (yes or no);
- Sexually transmitted infections (STIs): medical diagnosis of a sexually transmitted disease/infection in the last 12 months (yes or no)

While the Yellow and Indigenous skin colors categories are included in the total, due to the small number of observations (representing 1.36% of the population studied) and a high coefficient of variation, the IBGE does not recommend analyzing these data separately.

### Data Analysis

The descriptive analysis calculated the prevalence and respective 95% confidence intervals (95%CI) for some violence and its subtypes at the national level, by region and federative units. The proportions of the variables derived from the questions by subtype of violence were also calculated, and the prevalence rates were compared in the categories, with differences with non-overlapping 95%CI considered statistically significant.

The potential factors associated with VAW were assessed using the Poisson regression model with robust variance. Initially, a bivariate analysis was performed between each outcome variables and each explanatory variable to estimate the crude prevalence ratios (PRb). Variables with at least one category with a p-value < 0.20 were selected for inclusion in the multivariate model. These variables were added sequentially to the model, allowing the adjusted prevalence ratios (PRa) to be estimated. The final model was assessed at a 5% significance level.

The Software for Statistics and Data Science (Stata) version 14.0 was used to analyze the data through the survey module, which accounts for the effects of the sampling plan with weight previously defined by the IBGE.

### Ethical Aspects

This study used secondary data, exempting an ethics committee’s assessment. The PNS project was approved under Opinion No. 3,529,376, issued on August 23, 2019.

## RESULTS

**Figure 1.**
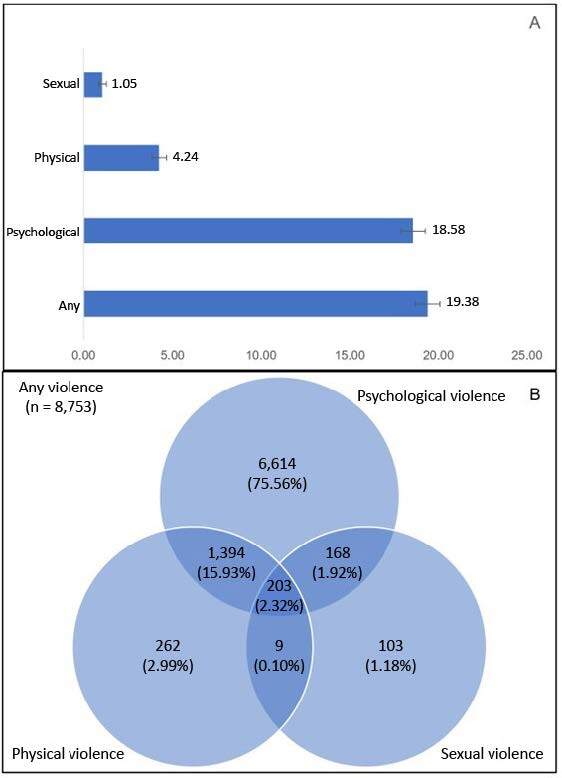
Prevalence (A) and intersection (B) of violence against women by subtype.

The prevalence of VAW was 19.38%. The most prevalent subtype was psychological (18.58%), followed by physical (4.24%) and sexual (1.05%) violence. Among the women exposed to violence, the majority reported psychological violence alone (75.56%). Additionally, psychological violence was the subtype most frequently associated with other subtypes of violence, whether physical (15.93%) or sexual (11.92%). Of the women exposed to violence, 20.27% reported experiencing at least two subtypes of violence (Figure 1).

**Figure 2.**
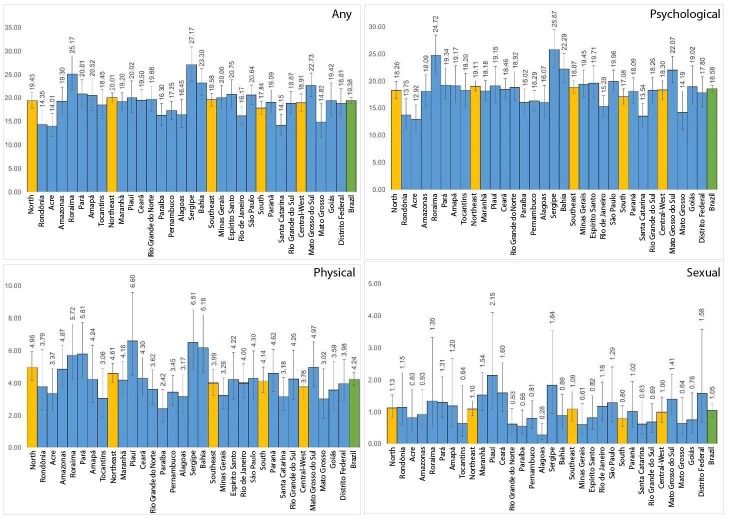
Prevalence of women’s exposure to violence and its subtypes, by Federative Unit and Region.

The Northeast includes the states with the highest prevalence of exposure to violence: Sergipe had the highest prevalence of any violence (27.17%) and psychological violence (25.87%), while Piauí had the highest prevalence of physical (6.60%) and sexual (2.15%) violence. Conversely, the North and Northeast also contains states with the lowest prevalence rates: Acre had the lowest prevalence of exposure to any violence (14.01%) and psychological violence (12.92%), Paraíba recorded the lowest prevalence of physical violence (2.42%) and Alagoas, of sexual violence (0.28%) (Figure 2).

In all subtypes of violence, the intimate partner emerged as the primary aggressor (31.98% for psychological, 52.37% for physical, and 53.28% for sexual), with the residence being the most common place of occurrence (55.32%, 72.79%, and 61.63% for psychological, physical, and sexual, respectively). Regarding the consequences, the majority of women exposed to violence reported psychological impacts (56.88%, 86.35%, and 72.60% of women exposed to psychological, physical, and sexual violence, respectively). Physical injuries were mentioned by 36.84% of women exposed to physical violence and 21.25% of those exposed to sexual violence. Sexual violence also resulted in sexual consequences for 5.29% of exposed women. Concerning healthcare utilization, 16.98% of women exposed to psychological violence, 18.54% of those exposed to physical violence, and 21.95% of those exposed to sexual violence sought health services, with hospitalizations longer than 24 hours required for 3.90%, 6.50%, and 2.88% of these cases, respectively (Table 1).

Women aged 18 to 24 years had higher prevalences of exposure to violence (30.56% for any violence, 29.08% for psychological violence, and 2.24% for sexual violence), as did those with a household income of up to 1 SM (21.16%, 20.26%, and 5.36%, for any violence, psychological violence, and physical violence, respectively). This pattern of VAW prevalence was also observed among women who reported alcohol consumption (31.26% for any violence, 29.86% for psychological violence, 10.35% for physical violence, and 2.83% for sexual violence), depression (30.90%, 30.21%, 7.40%, and 2.15% for any violence, psychological violence, physical violence, and sexual violence, respectively), and diagnosis of STIs (43.74%, 42.68%, 13.79%, and 6.47%, for any violence, psychological violence, physical violence, and sexual violence, respectively) (Table 2).

**Table 1.** Characteristics associated with violence by subtype of violence. National Survey of Health, 2019.

**Variables**	**Psychological Violence**	**Physical Violence**	**Sexual Violence**
	**(n = 8,379)**	**(n = 1,868)**	**(n = 483)**
	**% (95%CI)**	**% (95%CI)**	**% (95%CI)**
**Aggressor**			
Intimate partner	31.98	52.37	53.28
	(29.89–34.13)	(47.31–57.38)	(43.41–62.91)
Family member	27.30	24.48	8.38
	(25.55–29.13)	(20.73–28.67)	(5.08–13.51)
Acquaintance	23.75	9.46	14.03
	(22.14–25.44)	(7.18–12.37)	(9.55–20.14)
Others	16.97	13.69	24.31
	(15.56–18.47)	(11.04–16.84)	(17.95–32.04)
**Location**			
Residence	55.32	72.79	61.63
	(53.28–57.34)	(68.78–76.47)	(52.49–70.02)
Work/study location	15.49	5.39	10.63
	(14.08–17.01)	(3.90–7.40)	(6.22–17.55)
Public place	16.89	19.81	23.18
	(15.54–18.34)	(16.68–23.37)	(17.15–30.55)
Others	12.30	2.01	4.56
	(11.01–13.72)	(1.20–3.34)	(2.84–7.25)
**Recurrence**			
Yes	61.25	51.26	49.73
	(59.31–63.15)	(46.15–56.34)	(39.58–59.90)
No	38.75	48.74	50.27
	(36.85–40.69)	(43.66–53.85)	(40.10–60.42)
**Consequences**			
Psychological	56.88	86.35	72.60
	(54.75–58.99)	(83.54–88.74)	(64.89–79.16)
Physical	-	36.84	21.25
		(31.58–42.42)	(15.27–28.78)
Sexual	-	-	5.29
			(2.45–11.03)
**Healthcare**			
Search for healthcare	16.98	18.54	21.95
	(15.25–18.86)	(15.60–21.88)	(14.49–31.81)
Need for hospitalization	3.90	6.50	2.88
	(2.26–6.64)	(3.11–13.09)	(1.12–7.21)

95%CI: 95% confidence interval.

**Source:** Instituto Brasileiro de Geografia e Estatística.

**Table 2.** Prevalence of violence against women, according to potential associated factors. National Survey of Health, 2019.

**Characteristic**	**Any violence**	**Psychological Violence**	**Physical Violence**	**Sexual Violence**
	**Prevalence (95%CI)**	**Prevalence (95%CI)**	**Prevalence (95%CI)**	**Prevalence (95%CI)**
*Demographic*				
**Age range**				
18–24 years	30.56 (27.90–33.36)	29.08 (26.48–31.83)	7.89 (6.55–9.47)	2.24 (1.64–3.05)
25–39 years	22.58	21.69	5.74	1.09
	(21.33–23.89)	(20.44–22.98)	(5.01–6.57)	(0.85–1.39)
40–59 years	18.10	17.32	3.44	1.07
	(17.12–19.12)	(16.36–18.33)	(2.93–4.03)	(0.68–1.68)
≥ 60 years	11.09	10.77	1.54	0.29
	(10.16–12.10)	(9.84–11.77)	(1.20–1.98)	(0.16–0.51)
**Education**				
No education and incomplete primary education	16.87	16.12	3.94	0.94
	(15.88–17.90)	(15.16–17.14)	(3.38–4.58)	(0.56–1.59)
Complete primary education and incomplete secondary education	21.84	21.00	6.83	1.13
	(20.03–23.78)	(19.21–22.91)	(5.60–8.31)	(0.74–1.72)
Complete secondary education and incomplete higher education	20.96	20.07	4.15	1.23
	(19.71–22.28)	(18.83 – 21.37)	(3.65 – 4.73)	(0.96–1.57)
Higher education	19.18	18.52	2.98	0.80
	(17.61–20.86)	(16.96–20.18)	(2.15–4.11)	(0.57–1.12)
**Skin color**				
White	17.98	17.32	3.11	1.09
	(16.98–19.02)	(16.33–18.36)	(2.65–3.65)	(0.74–1.60)
Brown	20.21	19.36	5.17	1.01
	(19.22–21.23)	(18.40–20.36)	(4.57–5.84)	(0.82–1.25)
Black	21.28	20.09	5.29	1.04
	(19.45–23.24)	(18.28–22.04)	(4.32–6.48)	(0.71–1.52)
**Place of residence**				
Urban	19.82	18.99	4.31	1.03
	(19.07–20.60)	(18.26–19.75)	(3.87–4.79)	(0.82–1.30)
Rural	16.17	15.61	3.69	1.13
	(14.89–17.53)	(14.33–16.98)	(3.07–4.43)	(0.82–1.54)
*Socioeconomic*				
**Household income**				
Up to 1 MW	21.16	20.26	5.36	1.19
	(20.24–22.11)	(19.35–21.19)	(4.88–5.88)	(0.90–1.59)
More than 1 to 3 MW	17.56	16.87	3.20	0.93
	(16.35–18.84)	(15.68–18.14)	(2.45–4.18)	(0.69–1.26)
Above 3 MW	16.75	16.11	2.21	0.70
	(14.99–18.67)	(14.37–18.01)	(1.73–2.82)	(0.47–1.04)
**Marital status**				
Single	24.39	23.37	6.05	1.70
	(23.16–25.65)	(22.16–24.62)	(5.42–6.76)	(1.28–2.24)
Married	15.68	14.99	2.94	0.51
	(14.67–16.74)	(14.00–16.04)	(2.28–3.79)	(0.36–0.73)
Widowed	10.41	10.07	1.65	0.28
	(9.10–11.89)	(8.77–11.55)	(1.20–2.28)	(0.14–0.54)
Divorced, separated, or legally separated	23.92	23.11	4.78	1.40
	(21.91–26.06)	(21.11–25.23)	(3.84–5.95)	(0.90–2.17)
**Social support network**				
None	28.88	27.46	8.38	1.56
	(24.22–34.04)	(22.86–32.59)	(5.68–12.21)	(0.80–3.02)
1 person	26.76	25.65	7.42	1.97
	(23.85–29.88)	(22.78–28.75)	(5.77–9.50)	(1.21–3.19)
2 people	23.21	22.48	6.02	1.25
	(20.68–25.94)	(19.97–25.20)	(4.28–8.40)	(0.84–1.86)
3 or more people	18.37	17.60	3.79	0.96
	(17.65–19.12)	(16.89–18.33)	(3.42–4.19)	(0.75–1.23)
*Health*				
**Self-rated health**				
Very good or good	16.86	16.12	3.57	0.88
	(16.10–17.65)	(15.38–16.89)	(3.18–4.00)	(0.65–1.19)
Fair	25.11	24.06	5.41	1.36
	(23.75–26.51)	(22.73–25.45)	(4.65–6.28)	(1.04–1.78)
Poor or very poor	28.53	28.02	8.18	1.92
	(25.49–31.77)	(24.99–31.26)	(6.23–10.66)	(1.22–3.02)
**Alcohol consumption**				
Yes	31.26	29.86	10.35	2.83
	(28.08–34.63)	(26.70–33.22)	(8.61–12.39)	(1.93–4.13)
No	18.73	17.96	3.90	0.95
	(18.04–19.43)	(17.29–18.66)	(3.50–4.34)	(0.75–1.19)
**Depression**				
Yes	30.90	30.21	7.40	2.15
	(29.03–32.84)	(28.35–32.15)	(6.19–8.83)	(1.65–2.81)
No	17.39	16.57	3.69	0.85
	(16.67–18.13)	(15.87–17.30)	(3.34–4.07)	(0.65–1.12)
**Sexually transmitted infections**				
Yes	43.74	42.68	13.79	6.47
	(34.31–53.64)	(33.32–52.59)	(8.63–21.30)	(3.53–11.58)
No	19.20	18.41	4.17	1.01
	(18.52–19.91)	(17.74–19.27)	(3.77–4.60)	(0.81–1.25)

95%CI: 95% confidence interval; MW: minimum wage.

In the analysis of prevalence ratios in the multivariate model, higher prevalences of all subtypes of violence were observed among women aged 18 to 24 years (psychological violence: RPa 2.50; physical violence: RPa 4.90; sexual violence: RPa 4.72), 25 to 39 years (psychological violence: RPa 1.93; physical violence: RPa 3.77; sexual violence: RPa 2.78) and 40 to 59 years (psychological violence: RPa 1.47; physical violence: RPa 2.07; sexual violence: RPa 2.90) compared with women aged 60 years or older; who self-rated their health as fair (psychological violence: RPa 11.50; physical violence: RPa 1.46; sexual violence: RPa 1.50) and poor or very poor (psychological violence: RPa 1.68; physical violence: RPa 2.01; sexual violence: RPa 1.96), compared with women who self-rated their health as very good or good; among women with alcohol consumption (psychological violence: RPa 1.37; physical violence: RPa 1.91; sexual violence: RPa 2.14), depression (psychological violence: RPa 1.69; physical violence: RPa 1.96; sexual violence: RPa 2.35) and STIs (psychological violence: RPa 1.55; physical violence: RPa 1.82; sexual violence: RPa 3.47) (Table 3).

Women with no education and incomplete primary education (RPa: 1.53) or complete primary education and incomplete secondary education (RPa:1.71) had higher prevalences of physical violence than women with higher education, as did Black (RPa: 1.44) and Brown (RPa: 1.40) women compared with White women. Women living in rural areas showed lower prevalences of psychological (RPa: 0.83) and physical (RPa: 0.77) violence than those living in urban areas (Table 3).

**Table 3.** Crude and adjusted prevalence ratio (95%CI) of violence against women, according to potential associated factors. National Survey of Health, 2019.

**Variables**	**Any violence**		**Psychological violence**		**Physical violence**		**Sexual violence**	
	**RPb (95%CI)**	RPa* (95%CI)	**RPb (95%CI)**	RPa* (95%CI)	**RPb (95%CI)**	RPa* (95%CI)	**RPb (95%CI)**	RPa* (95%CI)
*Demographic*								
**Age range**								
18–24 years	2.76 (2.43–3.12)	2.55 (2.23–2.92)	2.70 (2.38–3.07)	2.50 (2.18–2.88)	5.12 (3.78–6.93)	4.90 (3.17–7.56)	7.75(4.07–14.74)	4.72(2.04–10.90)
25–39 years	2.04 (1.84–2.26)	1.95 (1.74–2.19)	2.01 (1.81–2.24)	1.93 (1.72–2.17)	3.72 (2.80–4.96)	3.77 (2.59–5.51)	3.77 (2.04–6.96)	2.78 (1.32–5.86)
40–59 years	1.63 (1.47–1.81)	1.49 (1.34–1.66)	1.61 (1.45–1.79)	1.47 (1.32–1.64)	2.23 (1.65–3.01)	2.07 (1.45–2.93)	3.69 (1.79–7.62)	2.90 (1.36–6.18)
≥ 60 years	1.00 (-)	1.00 (-)	1.00 (-)	1.00 (-)	1.00 (-)	1.00 (-)	1.00 (-)	1.00 (-)
**Education**								
No education and incomplete primary education	0.88 (0.80–0.97)		0.87 (0.79–0.96)		1.32 (0.99–1.76)	1.53 (1.14–2.05)	1.18 (0.63–2.20)	
Complete primary education and incomplete secondary education	1.14 (1.01–1.29)		1.13 (1.00–1.28)		2.30 (1.57–3.36)	1.71 (1.17–2.48)	1.41 (0.82–2.42)	
Complete secondary education and incomplete higher education	1.09 (0.98–1.22)		1.08 (0.97–1.21)		1.39 (0.98–1.99)		1.54 (1.01–2.34)	
Higher education	1.00 (-)		1.00 (-)		1.00 (-)	1.00 (-)	1.00 (-)	
**Skin color**								
White	1.00 (-)		1.00 (-)		1.00 (-)	1.00 (-)	1.00 (-)	
Brown	1.12 (1.04–1.21)		1.12 (1.04–1.21)		1.66 (1.40–1.97)	1.40(1.18 – 1.66)	0.93 (0.60–1.42)	
Black	1.18 (1.07–1.31)		1.16 (1.04–1.29)		1.70 (1.31–2.21)	1.44(1.12 – 1.85)	0.95 (0.55–1.64)	
**Place of residence**								
Urban	1.00 (-)	1.00 (-)	1.00 (-)	1.00 (-)	1.00 (-)	1.00 (-)	1.00 (-)	
Rural	0.82 (0.75–0.89)	0.82 (0.75–0.89)	0.82 (0.75–0.90)	0.83 (0.76–0.90)	0.86 (0.69–1.06)	0.77 (0.62–0.97)	1.09 (0.74–1.61)	
*Socioeconomic*								
**Household income**								
Up to 1 MW	1.26 (1.12–1.42)		1.26 (1.11–1.42)		2.43 (1.86–3.16)		1.70 (1.04–2.77)	
More than 1 to 3 MW	1.05 (0.92–1.20)		1.05 (0.91–1.20)		1.45 (1.01–2.09)		1.33 (0.81–2.18)	
Above 3 MW	1.00 (-)		1.00 (-)		1.00 (-)		1.00 (-)	
**Marital status**								
Single	1.00 (-)	1.00 (-)	1.00 (-)	1.00 (-)	1.00 (-)	1.00 (-)	1.00 (-)	1.00 (-)
Married	0.64 (0.59–0.70)	0.79 (0.72–0.86)	0.64 (0.59–0.70)	0.78 (0.71–0.86)	0.49 (0.37–0.64)	0.71 (0.54–0.94)	0.30 (0.19–0.47)	0.39 (0.21–0.70)
Widowed	0.43 (0.37–0.49)	0.69 (0.59–0.80)	0.43 (0.37–0.50)	0.69 (0.59–0.81)	0.27 (0.19–0.38)	0.64 (0.42–0.98)	0.16 (0.08–0.34)	0.35 (0.14–0.89)
Divorced, separated, or legally separated	0.98 (0.89–1.09)	1.15 (1.04–1.28)	0.99 (0.89–1.10)	1.16 (1.04–1.29)	0.79 (0.62–1.01)		0.82 (0.49–1.40)	
**Social support network**								
None	1.57 (1.32–1.87)	1.36 (1.15–1.62)	1.56 (1.30–1.87)	1.35 (1.13–1.61)	2.21 (1.49–3.30)	1.57 (1.04–2.37)	1.63 (0.80–3.29)	
1 person	1.46 (1.29–1.65)	1.28 (1.13–1.45)	1.46 (1.28–1.65)	1.28 (1.13–1.45)	1.96 (1.49–2.58)	1.44 (1.09–1.90)	2.05 (1.19–3.53)	
2 people	1.26 (1.12–1.42)		1.28 (1.13–1.44)		1.59 (1.15–2.20)		1.30 (0.82–2.08)	
3 or more people	1.00 (-)	1.00 (-)	1.00 (-)	1.00 (-)	1.00 (-)	1.00 (-)	1.00 (-)	
*Health*								
**Self-rated health**								
Very good or good	1.00 (-)	1.00 (-)	1.00 (-)	1.00 (-)	1.00 (-)	1.00 (-)	1.00 (-)	1.00 (-)
Fair	1.49 (1.39–1.60)	1.51 (1.41–1.61)	1.49 (1.39–1.60)	1.50 (1.40–1.61)	1.51 (1.29–1.78)	1.46 (1.24–1.72)	1.54 (1.03–2.30)	1.50 (1.01–2.23)
Poor or very poor	1.69 (1.51–1.90)	1.65 (1.48–1.84)	1.74 (1.54–1.96)	1.68 (1.50–1.88)	2.29 (1.72–3.06)	2.01(1.53–2 63)	2.18 (1.29–3.70)	1.96 (1.14–3.38)
**Alcohol consumption**								
Yes	1.67 (1.50–1.86)	1.38 (1.24–1.53)	1.66 (1.48–1.86)	1.37 (1.23–1.53)	2.65 (2.15–3.27)	1.91 (1.56–2.34)	2.99 (1.91–4.67)	2.14 (1.37–3.34)
No	1.00 (-)	1.00 (-)	1.00 (-)	1.00 (-)	1.00 (-)	1.00 (-)	1.00 (-)	1.00 (-)
**Depression**								
Yes	1.78 (1.65–1.92)	1.65 (1.54–1.78)	1.82 (1.69–1.97)	1.69 (1.57–1.82)	2.01 (1.68–2.40)	1.96 (1.65–2.32)	2.52 (1.73–3.68)	2.35 (1.65–3.35)
No	1.00 (-)	1.00 (-)	1.00 (-)	1.00 (-)	1.00 (-)	1.00 (-)	1.00 (-)	1.00 (-)
**Sexually transmitted infections**								
Yes	2.28 (1.82–2.86)	1.53 (1.22–1.91)	2.32 (1.84–2.92)	1.55 (1.23–1.95)	3.31 (2.08–5.26)	1.82 (1.10–3.02)	6.43 (3.41–12.12)	3.47 (1.89–6.37)
No	1.00 (-)	1.00 (-)	1.00 (-)	1.00 (-)	1.00 (-)	1.00 (-)	1.00 (-)	1.00 (-)

*multivariate model

95%CI: 95% confidence interval; RPa: adjusted prevalence ratio; MW: minimum wage.

## DISCUSSION

Approximately one in five Brazilian women reported some type of violence in the 12 months prior to the interview, with psychological violence being the most prevalent subtype . Most women reported that the violence was perpetrated by an intimate partner at home and having suffered consequences, mainly of a psychological nature. About one-fifth of these women sought health services, with up to 6.50% requiring hospitalization for more than 24 horas. VAW was associated with demographic, socioeconomic, and health factors, highlighting its macrostructural relationship with society.

The prevalence of VAW in the 2019 PNS was 19.38%, similar to findings in other studies^8 , 9^. It is noteworthy that while the North and Northeast presented the highest prevalence of violence, these regions also had the lowest prevalence of violence. This discrepancy may reflect the sexist culture in these regions, which normalizes violence and makes it harder for women to recognize it. Additionally, these regions suffer from a lack of services, limited access to information and protection networks, which directly affect the quality of violence reports^10^. Previous studies^11 , 12^ have highlighted this subnational variation, with the highest rates of underreporting occurring in these regions^13^. Therefore, public policies must account for local specificities to improve the response to violence, considering the context and cultural nuances of each place.

Despite these concerning numbers, it is important to emphasize that the prevalence of violence may be even higher. Violence remains a stigmatized issue, and many women conceal their experiences, especially when questioned by strangers in a survey^14^. The normalization of abusive relationships and the difficulty in recognizing violence in everyday acts are additional barriers^14^. In this context, a survey conducted by DataSenado^15^ in 2023 revealed that when women were directly asked whether they had experienced any type of violence in the past 12 months, only 7.01% confirmed such an experience. However, when specific examples of violent acts were provided, the percentage of women reporting exposure to violence increased significantly: 30.27% reported exposure to psychological or moral violence and 8.13% mentioned physical violence.

It is worth highlighting the high prevalence of psychological violence, which is the subtype with the highest occurrence, both in isolation and concomitantly with other subtypes of violence. In fact, only 4.27% of women reported exclusively physical and/or sexual violence. Similar data were found in previous studies^10 , 14^. This finding shows that the domination of women often occurs on a symbolic level, being exercised through threats and humiliation^16^. Furthermore, it challenges the traditional view that tends to hierarchize the types of violence^14^. Psychological violence should not be underestimated nor seen as a prelude to “more serious” forms of violence, such as physical or sexual violence. This subtype of violence is, in itself, a serious violation of women’s dignity and health, causing persistent and profound emotional and mental impacts, a reality supported in this study by the high percentage of women who reported psychological consequences after exposure to violence.

This study demonstrated that the primary aggressor of women was an intimate partner and that the most common place where violence occurred was the home, highlighting the fallacy of the protective family and the supposed safety of the home, while reaffirming the role of the patriarchal system at the root of VAW^2^. Within this system, violence emerges as a mean of maintaining the supposed supremacy of men, sexualizing positions of power, and restricting access to rights by different social groups^17^. Therefore, it is essential to approach the phenomenon of VAW in a broad context, recognizing the structures of inequality that influence its occurrence and the social and cultural dynamics that perpetuate gender inequities.

Most women reported consequences resulting from violence, especially psychological ones, but only one-fifth of them sought healthcare. Among these women, those who suffered sexual and physical violence were the ones who sought healthcare the most, with the latter being the group with the greatest need for hospitalization. This pattern suggests that Brazilian women still see the health sector primarily as a place to address the direct consequences of violence, such as treating injuries and providing prophylaxis of STIs, rather than as a gateway to the Care Network for women who live with violence^13^.

This study confirmed the correlation between demographic and socioeconomic factors and VAW. Younger women, those with less education, Black and Brown women, and those with a smaller support network had a higher prevalence of violence. These factors are often associated with restricted access to the labor market^18^, which can exacerbate their economic vulnerability and financial dependence^19^. Although VAW does not directly derive from poverty, it is necessary to recognize the stress faced by economically vulnerable population. These individuals encounter worse job opportunities, less access to public policies, and a devaluation of their purchasing power, all of which negatively affect individual and community well-being^20^. The stress caused by social inequities increases the likelihood of violence being used to resolve conflicts^21^, and women’s economic disadvantages prevent them from empowering themselves and breaking free from violent relationships^17^. Therefore, effective Public Policies to address VAW must promote female autonomy through broad access to education, income, and housing, among others, to minimize exposure to violence.

Women living in rural areas had lower prevalence rates of exposure to violence. Some studies attribute the higher prevalence of violence in cities to the country’s exclusionary urbanization process, which increases social inequalities^22^. However, attention must be paid to the silencing and invisibility of women in rural areas. An integrative review^23^ showed that these women are highly exposed to violence, particularly domestic violence and that such acts occur frequently. However, cultural and religious issues often prevent these women from breaking the silence, and it can take many years for women living in rural areas to leave abusive relationships due to a lack of knowledge or access to public policies for empowerment and protection.

The study also highlighted the association between VAW and the health factors of Brazilian women. Violence is a risk factor for anxiety and depression, which can influence the poor perception of one’s health^24^. Additionally, violence directly contributes to physical sequelae and STIs, compromising the quality of life of women who live with it^25^. Data from the Global Burden of Disease^26^ (GBD) showed that, for Brazilian women in 2019, interpersonal violence was responsible for a rate of 44.65 years lived with disabilities, with intimate partner violence contributing to 7.11% of depressive disorders and 7.99% of HIV/AIDS cases. As for alcohol consumption, alcoholic women are known to be more vulnerable, often engaging in prostitution and violence to sustain their addiction^27^. Moreover, alcohol plays a role in the imbalance of relationships and can precipitate family conflicts^19^.

The data found in this study show a strong association between the experience of violence and factors that make the population vulnerable. It is essential to highlight that Brazil underwent a period of austerity, during which the limitation of social spending exacerbated the inequalities present in the country. The dismantling of public policies implemented between 2016 and 2022 affected progress in advancing gender equality, with a reduced budget for legal and assistance support services for women^28^. Additionally, measures taken during these years facilitated access to weapons, putting women’s lives at greater risk, especially those living with violence^29^.

This study analyzed data from the year before the COVID-19 pandemic. Social restrictions linked to health recommendations imposed new family dynamics, making women who lived with violence even more vulnerable since interaction with their likely aggressors increased, as did household economic stress^30^. A previous study showed that, despite a reduction in reports, VAW was impacted by the pandemic, with an increase in telephone reports and requests for protective measures^30^. Thus, data from the 2019 PNS serve as a baseline for future analyses on the prevalence of VAW during and after the global health crisis.

One limitation of this study is the restriction of the questions in the violence module to women over 18 years old, which prevents an understanding of the issue in younger age groups. The PNS also does not cover homeless populations, nursing homes, *quilombos*, and villages and is limited to evaluating only three subtypes of violence, excluding patrimonial, moral, or negligence violence, for example. These exclusions lead to an underestimation of the prevalence, contributing to the invisibility of the problem, especially among women in more vulnerable situations. Moreover, the focus on the characteristics of violence is limited to the most severe event reported in the past 12 months, restricting the understanding of all the episodes of violence experienced by women. To reduce the underestimation of prevalence due to the stigma of violence, efforts were made to ensure the privacy of women when answering the questionnaire, distancing them from their possible aggressor, and allowing direct response on the device, reducing possible embarrassment, even with interviewers trained for the context.

In conclusion, VAW is highly prevalent in Brazil, with approximately one-fifth of the female population reporting at least one episode of violence in the past 12 months. This issue is associated with macro-structural factors in society, such as racism and sexism, directly impacting women’s autonomy by causing long-term consequences and diminishing their quality of life. These results highlight the relationship between health inequities and social vulnerabilities, intensifying the need for coordinated efforts across various sectors of society to develop Public Policies that ensure the protection of women’s lives and rights.
