# Supplementary material for: Who are the adult women exposed to violence in Brazil?
Source: Rev Saude Publica. 2025 Apr 7;59:e8. doi: 10.11606/s1518-8787.2025059005701 (PMC11978288; doi:10.11606/s1518-8787.2025059005701)
Supplement: Translation

# Translation: Quem são as mulheres adultas expostas à violência no Brasil?

**Authors:** Nádia Machado de Vasconcelos, Crizian Saar Gomes, Juliana Bottoni de Souza, Fabiana Martins Dias de Andrade, Regina Tomie Ivata Bernal, Elaine Leandro Machado, Adalgisa Peixoto Ribeiro, Deborah Carvalho Malta

**Affiliations:** I Universidade Federal de Minas Gerais. Faculdade de Medicina. Programa de Pós-Graduação em Saúde Pública. Belo Horizonte, MG, Brasil; II Universidade Federal de Minas Gerais. Escola de Enfermagem. Observatório de Doenças e Agravos Não Transmissíveis. Belo Horizonte, MG, Brasil; III Universidade Federal de Minas Gerais. Escola de Enfermagem. Programa de Pós-Graduação em Enfermagem. Belo Horizonte, MG, Brasil; IV Universidade Federal de Minas Gerais. Faculdade de Medicina. Departamento de Medicina Preventiva e Social. Belo Horizonte, MG, Brasil; V Universidade Federal de Minas Gerais. Escola de Enfermagem. Departamento de Enfermagem Materno Infantil e Saúde Pública. Belo Horizonte, MG, Brasil

**Keywords:** Violência de Gênero, Violência Contra as Mulheres, Estudos Transversais, Epidemiologia, Brazil

## Abstract

Estimar a prevalência de subtipos da violência e analisar alguns dos fatores demográficos, socioeconômicos e de saúde associados a Violência Contra as Mulheres (VCM) no Brasil.

Estudo epidemiológico transversal utilizando base de dados da Pesquisa Nacional de Saúde (PNS) 2019. Foram estimadas as prevalências de alguns subtipos da violência (psicológica, física e sexual) nos 12 meses anteriores a entrevista, no Brasil e nas unidades federativas. Analisou-se também algumas das características de cada subtipo. Estimou-se ainda a Razão de Prevalência bruta e ajustada por modelo multivariado segundo potenciais fatores demográficos, socioeconômicos e de saúde associados: faixa etária, escolaridade, cor da pele, local de moradia, renda domiciliar, estado civil, rede social de apoio, autoavaliação em saúde, consumo de álcool, depressão e infecções sexualmente transmissíveis (ISTs).

Em 2019, 19,38% das mulheres brasileiras relataram algum episódio de violência, sendo a violência psicológica o subtipo mais comum, tanto isolada quanto concomitante com outros subtipos. O principal agressor foi um parceiro íntimo e a maioria dos atos violentos ocorreu na residência, com mais da metade das mulheres relatando ao menos uma consequência desses atos. Mulheres mais jovens, com pior autoavaliação em saúde, consumo de álcool, depressão e diagnóstico de ISTs tiveram maior prevalência de todos os subtipos de violência.

Uma em cada cinco mulheres brasileiras relataram algum episódio de violência nos últimos 12 meses. A VCM está positivamente associada a idades mais jovens, baixa escolaridade, cor da pele preta e parda, menor rede de apoio, além de fatores ligados a saúde, como autoavaliação em saúde, consumo de álcool, depressão e ISTs.

## INTRODUÇÃO

A Violência Contra as Mulheres (VCM), definida como “qualquer ação ou conduta, baseada no gênero, que cause morte, dano ou sofrimento físico, sexual ou psicológico à mulher, tanto no âmbito público como no privado”^1^, constitui um problema social e de Saúde Pública. É um fenômeno multicausal e associado às desigualdades econômicas e a aspectos subjetivos e comportamentais vigentes na sociedade^2^.

Globalmente, uma a cada três mulheres acima de 15 anos já sofreu violência física e/ou sexual ao longo da vida, com prevalência semelhante nas Américas^3^. No Brasil, 35,60% dos homicídios femininos registrados em 2022 foram considerados feminicídios, a forma letal da VCM^4^.

Mulheres expostas à VCM têm maior probabilidade de desenvolver transtornos mentais, comportamentos de risco e de contaminação por infecções sexualmente transmissíveis (IST)^1^. Ademais, a VCM impacta significativamente a economia nacional, com estudo anterior mostrando que, em dez anos, essa violência reduziu o Produto Interno Bruto brasileiro em 214,42 bilhões de reais^5^.

A VCM está relacionada a desigualdades sociais e os baixos níveis de escolaridade e de condição econômica podem influenciar sua ocorrência^2^. Porém, o não reconhecimento da violência por parte das mulheres e a falta de treinamento adequado aos profissionais de saúde para identificar os sinais indiretos desse agravo levam à subnotificação da VCM, o que dificulta a coleta de dados e limita o conhecimento da realidade desse problema no país^6^.

No Brasil, há uma lacuna de estudos com abrangência nacional que tenham estimado a VCM e seus subtipos, assim como os potenciais fatores associados. Nesse sentido, o objetivo deste estudo foi estimar a prevalência de subtipos da violência e analisar alguns dos fatores demográficos, socioeconômicos e de saúde associados a VCM no Brasil.

Entender a complexa interação da VCM com outros fatores presentes na população em âmbito nacional torna-se fundamental para o planejamento e a implementação de Políticas Públicas voltadas para mulheres em situação de violência, com especial atenção àquelas em vulnerabilidade social.

## MÉTODOS

### Delineamento e Fonte de Dados

Estudo epidemiológico transversal, de caráter analítico, utilizando dados da Pesquisa Nacional de Saúde (PNS), realizada em 2019.

A PNS é um inquérito domiciliar que utiliza amostra representativa da população brasileira, refletindo a distribuição por sexo, faixa etária, cor da pele, renda, macrorregião de moradia, entre outros. A amostra da PNS 2019 constituiu-se em um plano de conglomerado de três estágios de seleção: i) setores censitários ou conjunto de setores; ii) domicílios; e iii) moradores. Foram realizadas 90.846 entrevistas, o que correspondeu a 96,50% de taxa de respostas. Para o presente estudo, selecionou-se as mulheres com 18 anos ou mais de todo o Brasil que responderam ao módulo de violência (n = 46.869). Detalhes da metodologia da PNS 2019 estão em publicação anterior^7^.

### Variáveis

O questionário da PNS em 2019 investigou a exposição a três subtipos de violência, ao perguntar se, nos últimos 12 meses, alguém:

1. Te ofendeu, humilhou ou ridicularizou na frente de outras pessoas?; gritou com você ou te xingou?; usou redes sociais ou celular para ameaçar, ofender, xingar ou expor imagens suas sem o seu consentimento?; ameaçou verbalmente lhe ferir ou machucar alguém importante para você?; destruiu alguma coisa sua de propósito? (violência psicológica)
2. Te deu um tapa ou uma bofetada?; te empurrou, segurou com força ou jogou algo em você com a intenção de machucar?; te deu um soco, chutou ou arrastou pelo cabelo?; tentou ou efetivamente estrangulou, asfixiou ou te queimou de propósito?; te ameaçou ou feriu com uma faca, arma de fogo ou alguma outra arma ou objeto? (violência física)
3. Tocou, manipulou, beijou ou expôs partes do seu corpo contra sua vontade?; te ameaçou ou forçou a ter relações sexuais ou quaisquer outros atos sexuais contra sua vontade? (violência sexual)

Para esse estudo, considerou-se que a mulher estava exposta a um determinado subtipo de violência se ela confirmasse a ocorrência de pelo menos uma das situações previstas nesse subtipo. Adicionalmente, desenvolveu-se a variável “Alguma Violência”, a qual engloba a indicação de resposta afirmativa para pelo menos um dos subtipos de violência abordados.

Entre as mulheres que relataram violência, analisou-se as seguintes variáveis derivadas das perguntas por subtipo de violência:

- Agressor: parceiro íntimo; familiar; pessoa conhecida e outros
- Local: residência; local de trabalho/estudo; local público e outro
- Recorrência: sim ou não
- Consequências para a saúde: a) psicológicas: medo, tristeza, desânimo, dificuldades para dormir, ansiedade, depressão ou outras consequências psicológicas; b) físicas: hematomas, cortes, fraturas, queimaduras ou outras lesões físicas ou ferimentos; e c) sexuais: doença sexualmente transmissível ou gravidez indesejada.

Para mulheres que relataram consequências decorrentes da violência, analisou-se a procura de atendimento em saúde e a necessidade de internação por mais de 24 horas.

Para avaliar os potenciais fatores associados a VCM, foram selecionadas algumas variáveis explicativas relacionadas às características demográficas, socioeconômicas e de saúde das mulheres:

a. Demográficas:

- Faixa etária: 18 a 24 anos; 25 a 39 anos; 40 a 59 anos e 60 anos ou mais;
- Escolaridade: sem instrução e fundamental incompleto; fundamental completo e médio incompleto; médio completo e superior incompleto; e superior;
- Cor da pele: branca, parda e preta;
- Local de moradia: urbano e rural;

b. Socioeconômicas:

- Renda: até 1 salário-mínimo (SM); mais de 1 a 3 SM; acima de 3 SM;
- Estado civil: solteira; casada; viúva e divorciada, desquitada ou separada judicialmente;
- Rede social de apoio: soma de pessoas com quem a mulher respondeu poder contar em momentos bons ou ruins (nenhum, um, dois e três ou mais);

c. Saúde:

- Autoavaliação em saúde: muito boa ou boa; regular; ruim ou muito ruim;
- Consumo de álcool: consumo de 8 doses ou mais de álcool por semana (sim ou não);
- Depressão: diagnóstico de depressão por médico ou profissional de saúde mental (sim ou não);
- Infecções sexualmente transmissíveis (ISTs): diagnóstico médico, nos últimos 12 meses, de doença / infecção sexualmente transmissível (sim ou não)

As cores da pele amarela e indígena estão contidas no total, porém, devido ao pequeno número de observações (representando, conjuntamente, 1,36% da população estudada) e elevado coeficiente de variação, o IBGE não recomenda a análise de seus dados discriminadamente.

### Análise de Dados

Na análise descritiva, calculou-se as prevalências e seus respectivos intervalos de confiança de 95% (IC95%) para alguma violência e seus subtipos, a nível nacional, por região e unidades federativas. Calculou-se ainda as proporções das variáveis derivadas das perguntas por subtipo de violência e realizou-se a comparação das prevalências nas categorias, sendo consideradas como estatisticamente significativas as diferenças em que não houve sobreposição dos IC95%.

Os potenciais fatores associados à VCM foram avaliados a partir do modelo de regressão de Poisson com variância robusta. Inicialmente, conduziu-se uma análise bivariada entre as variáveis de desfecho e cada variável explicativa, sendo estimadas as razões de prevalência brutas (RPb). Para a construção do modelo multivariado, selecionaram-se as variáveis que apresentaram pelo menos uma categoria com valor-p < 0,20. Essas variáveis foram adicionadas sequencialmente ao modelo, permitindo a estimativa das Razões de Prevalência ajustada (RPa). O modelo final foi considerado ao nível de significância de 5%.

Utilizou-se o *Software for Statistics and Data Science* (*Stata)* versão 14.0 para análise dos dados por meio do módulo *survey*, que considera efeitos do plano amostral, com peso previamente definido pelo IBGE.

### Aspectos Éticos

O presente estudo utilizou dados secundários, dispensando apreciação por comitê de ética em pesquisa. O projeto da PNS foi aprovado sob o Parecer nº 3.529.376, emitido em 23 de agosto de 2019.

## RESULTADOS

A prevalência da VCM foi de 19,38%. O subtipo mais prevalente foi a violência psicológica (18,58%), seguida pela física (4,24%) e sexual (1,05%). Dentre as mulheres expostas a violência, a maioria relatou violência psicológica isoladamente (75,56%). Além disso, esse também foi o subtipo mais frequentemente associado a outros subtipos de violência, seja a violência física (15,93%) ou sexual (1,92%). Das mulheres expostas a violência, 20,27% relataram ter sofrido pelo menos dois subtipos de violência (Figura 1).

**Figura 1.** Prevalência (A) e intersecção (B) da violência contra as mulheres, por subtipo.

O Nordeste contém os estados com maiores prevalências de exposição a violência: Sergipe apresentou a maior prevalência para alguma violência (27,17%) e violência psicológica (25,87%); enquanto o Piauí foi o estado com maiores prevalências de violência física (6,60%) e sexual (2,15%). Por outro lado, Norte e Nordeste abrigam os estados com as menores prevalências: o Acre apresentou as menores prevalências de exposição a alguma violência (14,01%) e violência psicológica (12,92%), a Paraíba registrou a menor prevalência de violência física (2,42%) e Alagoas, da violência sexual (0,28%) (Figura 2).

**Figura 2.** Prevalência da exposição das mulheres a violência e seus subtipos, por Unidade Federativa e Região.

Em todos os subtipos de violência, o parceiro íntimo apareceu como principal agressor (31,98% para psicológica; 52,37% para física e 53,28% para sexual), sendo a residência o local mais comum de ocorrência (55,32%, 72,79% e 61,63% para psicológica, física e sexual, respectivamente). Em relação às consequências, a maioria das mulheres expostas a violência relatou impactos psicológicos (56,88%, 86,35% e 72,60% das mulheres que sofreram violência psicológica, física e sexual, respectivamente), enquanto lesões físicas foram mencionadas por 36,84% das mulheres que sofreram violência física e 21,25% das que sofreram violência sexual. A violência sexual também resultou em consequências sexuais para 5,29% das mulheres expostas. Quanto à busca por atendimento em saúde, 16,98% das mulheres expostas a violência psicológica, 18,54% das expostas a violência física e 21,95% das expostas a violência sexual procuraram serviços de saúde, com internações superiores a 24 horas necessárias para 3,90%, 6,50% e 2,88% desses casos, respectivamente (Tabela 1).

Mulheres de 18 a 24 anos apresentaram maiores prevalências de exposição a violência (30,56% para alguma violência, 29,08% para violência psicológica e 2,24% para violência sexual), assim como aquelas com renda domiciliar de até 1SM (21,16%, 20,26% e 5,36%, para alguma violência, violência psicológica e violência física, respectivamente). Esse padrão de prevalência da VCM também foi observado entre as mulheres que relataram consumo de álcool (31,26% para alguma violência, 29,86% para violência psicológica, 10,35% para violência física e 2,83% para violência sexual), depressão (30,90%, 30,21%, 7,40% e 2,15% para alguma violência, violência psicológica, violência física e violência sexual, respectivamente) e diagnóstico de ISTs (43,74%, 42,68%, 13,79% e 6,47%, para alguma violência, violência psicológica, violência física e violência sexual, respectivamente) (Tabela 2).

**Tabela 1.** Características associadas à violência, por subtipo de violência. Pesquisa Nacional de Saúde, 2019.

**Variáveis**	**Violência Psicológica**	**Violência Física**	**Violência Sexual**
	**(n = 8.379)**	**(n = 1.868)**	**(n = 483)**
	**% (IC95%)**	**% (IC95%)**	**% (IC95%)**
**Agressor**			
Parceiro íntimo	31,98 (29,89–34,13)	52,37 (47,31–57,38)	53,28 (43,41–62,91)
Familiar	27,30 (25,55–29,13)	24,48 (20,73–28,67)	8,38 (5,08–13,51)
Pessoa conhecida	23,75 (22,14–25,44)	9,46 (7,18–12,37)	14,03 (9,55–20,14)
Outros	16,97 (15,56–18,47)	13,69 (11,04–16,84)	24,31 (17,95–32,04)
**Local**			
Residência	55,32 (53,28–57,34)	72,79 (68,78–76,47)	61,63 (52,49–70,02)
Local de trabalho/estudo	15,49 (14,08–17,01)	5,39 (3,90–7,40)	10,63 (6,22–17,55)
Local público	16,89 (15,54–18,34)	19,81 (16,68–23,37)	23,18 (17,15–30,55)
Outros	12,30 (11,01–13,72)	2,01 (1,20–3,34)	4,56 (2,84–7,25)
**Recorrência**			
Sim	61,25 (59,31–63,15)	51,26 (46,15–56,34)	49,73 (39,58–59,90)
Não	38,75 (36,85–40,69)	48,74 (43,66–53,85)	50,27 (40,10–60,42)
**Consequências**			
Psicológicas	56,88 (54,75–58,99)	86,35 (83,54–88,74)	72,60 (64,89–79,16)
Físicas	-	36,84 (31,58–42,42)	21,25 (15,27–28,78)
Sexuais	-	-	5,29 (2,45–11,03)
**Atendimento em saúde**			
Procura por atendimento em saúde	16,98 (15,25–18,86)	18,54 (15,60–21,88)	21,95 (14,49–31,81)
Necessidade de internação	3,90 (2,26–6,64)	6,50 (3,11–13,09)	2,88 (1,12–7,21)

IC95%: intervalo de confiança de 95%.

Fonte: Instituto Brasileiro de Geografia e Estatística.

**Tabela 2.** Prevalência da violência contra as mulheres, segundo potenciais fatores associados. Pesquisa Nacional de Saúde, 2019.

**Variáveis**	**Alguma violência**	**Violência Psicológica**	**Violência Física**	**Violência Sexual**
	**Prevalência (IC95%)**	**Prevalência (IC95%)**	**Prevalência (IC95%)**	**Prevalência (IC95%)**
Demográficas				
**Faixa etária**				
18–24 anos	30,56 (27,90–33,36)	29,08 (26,48–31,83)	7,89 (6,55–9,47)	2,24 (1,64–3,05)
25–39 anos	22,58 (21,33–23,89)	21,69 (20,44–22,98)	5,74 (5,01–6,57)	1,09 (0,85–1,39)
40–59 anos	18,10 (17,12–19,12)	17,32 (16,36–18,33)	3,44 (2,93–4,03)	1,07 (0,68–1,68)
≥ 60 anos	11,09 (10,16–12,10)	10,77 (9,84–11,77)	1,54 (1,20–1,98)	0,29 (0,16–0,51)
**Escolaridade**				
Sem instrução e fundamental incompleto	16,87 (15,88–17,90)	16,12 (15,16–17,14)	3,94 (3,38–4,58)	0,94 (0,56–1,59)
Fundamental completo e médio incompleto	21,84 (20,03 – 23,78)	21,00 (19,21 – 22,91)	6,83 (5,60 – 8,31)	1,13 (0,74 – 1,72)
Médio completo e superior incompleto	20,96 (19,71 – 22,28)	20,07 (18,83 – 21,37)	4,15 (3,65 – 4,73)	1,23 (0,96 – 1,57)
Superior completo	19,18 (17,61–20,86)	18,52 (16,96–20,18)	2,98 (2,15–4,11)	0,80 (0,57–1,12)
**Cor da pele**				
Branca	17,98 (16,98–19,02)	17,32 (16,33–18,36)	3,11 (2,65–3,65)	1,09 (0,74–1,60)
Parda	20,21 (19,22–21,23)	19,36 (18,40–20,36)	5,17 (4,57–5,84)	1,01 (0,82–1,25)
Preta	21,28 (19,45–23,24)	20,09 (18,28–22,04)	5,29 (4,32–6,48)	1,04 (0,71–1,52)
**Local de moradia**				
Urbano	19,82 (19,07–20,60)	18,99 (18,26–19,75)	4,31 (3,87–4,79)	1,03 (0,82–1,30)
Rural	16,17 (14,89–17,53)	15,61 (14,33–16,98)	3,69 (3,07–4,43)	1,13 (0,82–1,54)
Socioeconômicas				
**Renda domiciliar**				
Até 1 SM	21,16 (20,24–22,11)	20,26 (19,35–21,19)	5,36 (4,88–5,88)	1,19 (0,90–1,59)
Mais de 1 até 3 SM	17,56 (16,35–18,84)	16,87 (15,68–18,14)	3,20 (2,45–4,18)	0,93 (0,69–1,26)
Acima de 3 SM	16,75 (14,99–18,67)	16,11 (14,37–18,01)	2,21 (1,73–2,82)	0,70 (0,47–1,04)
**Estado Civil**				
Solteira	24,39 (23,16–25,65)	23,37 (22,16–24,62)	6,05 (5,42–6,76)	1,70 (1,28–2,24)
Casada	15,68 (14,67–16,74)	14,99 (14,00–16,04)	2,94 (2,28–3,79)	0,51 (0,36–0,73)
Viúva	10,41 (9,10–11,89)	10,07 (8,77–11,55)	1,65 (1,20–2,28)	0,28 (0,14–0,54)
Divorciada, desquitada ou separada judicialmente	23,92 (21,91–26,06)	23,11 (21,11–25,23)	4,78 (3,84–5,95)	1,40 (0,90–2,17)
**Rede de apoio social**				
Nenhuma	28,88 (24,22–34,04)	27,46 (22,86–32,59)	8,38 (5,68–12,21)	1,56 (0,80–3,02)
1 pessoa	26,76 (23,85–29,88)	25,65 (22,78–28,75)	7,42 (5,77–9,50)	1,97 (1,21–3,19)
2 pessoas	23,21 (20,68–25,94)	22,48 (19,97–25,20)	6,02 (4,28–8,40)	1,25 (0,84–1,86)
3 ou mais pessoas	18,37 (17,65–19,12)	17,60 (16,89–18,33)	3,79 (3,42–4,19)	0,96 (0,75–1,23)
Saúde				
**Autoavaliação em saúde**				
Muito boa ou boa	16,86 (16,10–17,65)	16,12 (15,38–16,89)	3,57 (3,18–4,00)	0,88 (0,65–1,19)
Regular	25,11 (23,75–26,51)	24,06 (22,73–25,45)	5,41 (4,65–6,28)	1,36 (1,04–1,78)
Ruim ou muito ruim	28,53 (25,49–31,77)	28,02 (24,99–31,26)	8,18 (6,23–10,66)	1,92 (1,22–3,02)
**Consumo de álcool**				
Sim	31,26 (28,08–34,63)	29,86 (26,70–33,22)	10,35 (8,61–12,39)	2,83 (1,93–4,13)
Não	18,73 (18,04–19,43)	17,96 (17,29–18,66)	3,90 (3,50–4,34)	0,95 (0,75–1,19)
**Depressão**				
Sim	30,90 (29,03–32,84)	30,21 (28,35–32,15)	7,40 (6,19–8,83)	2,15 (1,65–2,81)
Não	17,39 (16,67–18,13)	16,57 (15,87–17,30)	3,69 (3,34–4,07)	0,85 (0,65–1,12)
**Infecções sexualmente transmissíveis**				
Sim	43,74 (34,31–53,64)	42,68 (33,32–52,59)	13,79 (8,63–21,30)	6,47 (3,53–11,58)
Não	19,20 (18,52–19,91)	18,41 (17,74–19,27)	4,17 (3,77–4,60)	1,01 (0,81–1,25)

IC95%: intervalo de confiança de 95%; SM: salário-mínimo.

Na análise das razões de prevalência no modelo multivariado foi possível observar maiores prevalências de todos os subtipos de violência entre mulheres de 18 a 24 anos (violência psicológica: RPa 2,50; violência física: RPa 4,90; violência sexual: RPa 4,72), de 25 a 39 anos (violência psicológica: RPa 1,93; violência física: RPa 3,77; violência sexual: RPa 2,78) e de 40 a 59 anos (violência psicológica: RPa 1,47; violência física: RPa 2,07; violência sexual: RPa 2,90) em relação as mulheres com 60 anos ou mais; que autoavaliaram a saúde como regular (violência psicológica: RPa 1,50; violência física: RPa 1,46; violência sexual: RPa 1,50) e ruim ou muito ruim (violência psicológica: RPa 1,68; violência física: RPa 2,01; violência sexual: RPa 1,96), em relação as mulheres que autoavaliaram a saúde como muito boa ou boa; entre as mulheres com consumo de álcool (violência psicológica: RPa 1,37; violência física: RPa 1,91; violência sexual: RPa 2,14), depressão (violência psicológica: RPa 1,69; violência física: RPa 1,96; violência sexual: RPa 2,35) e ISTs (violência psicológica: RPa 1,55; violência física: RPa 1,82; violência sexual: RPa 3,47) (Tabela 3).

As mulheres sem instrução e com fundamental incompleto (RPa: 1,53) ou com fundamental completo e médio incompleto (RPa:1,71) apresentaram maiores prevalências de violência física que as mulheres com ensino superior completo, assim como as mulheres pretas (RPa: 1,44) e pardas (RPa: 1,40), em relação às brancas. Já as mulheres que moravam na região rural mostraram menores prevalências de violência psicológica (RPa: 0,83) e física (RPa: 0,77) que aquelas moradoras da zona urbana (Tabela 3).

**Tabela 3.** Razão de Prevalência bruta e ajustada (IC95%) da violência contra mulheres, segundo potenciais fatores associados. Pesquisa Nacional de Saúde, 2019.

**Variáveis**	**Alguma violência**		**Violência psicológica**		**Violência física**		**Violência sexual**	
	**RPb (IC95%)**	RPaj* (IC95%)	**RPb (IC95%)**	RPaj* (IC95%)	**RPb (IC95%)**	RPaj* (IC95%)	**RPb (IC95%)**	RPaj* (IC95%)
Demográficas								
**Faixa etária**								
18–24 anos	2,76 (2,43–3,12)	2,55 (2,23–2,92)	2,70 (2,38–3,07)	2,50 (2,18–2,88)	5,12 (3,78–6,93)	4,90 (3,17–7,56)	7,75 (4,07–14,74)	4,72 (2,04–10,90)
25–39 anos	2,04 (1,84–2,26)	1,95 (1,74–2,19)	2,01 (1,81–2,24)	1,93 (1,72–2,17)	3,72 (2,80–4,96)	3,77 (2,59–5,51)	3,77 (2,04–6,96)	2,78 (1,32–5,86)
40–59 anos	1,63 (1,47–1,81)	1,49 (1,34–1,66)	1,61 (1,45–1,79)	1,47 (1,32–1,64)	2,23 (1,65–3,01)	2,07 (1,45–2,93)	3,69 (1,79–7,62)	2,90 (1,36–6,18)
≥ 60 anos	1,00 (-)	1,00 (-)	1,00 (-)	1,00 (-)	1,00 (-)	1,00 (-)	1,00 (-)	1,00 (-)
**Escolaridade**								
Sem instrução e fundamental incompleto	0,88 (0,80–0,97)		0,87 (0,79–0,96)		1,32 (0,99–1,76)	1,53 (1,14–2,05)	1,18 (0,63–2,20)	
Fundamental completo e médio incompleto	1,14 (1,01–1,29)		1,13 (1,00–1,28)		2,30 (1,57–3,36)	1,71 (1,17–2,48)	1,41 (0,82–2,42)	
Médio completo e superior incompleto	1,09 (0,98–1,22)		1,08 (0,97–1,21)		1,39 (0,98–1,99)		1,54 (1,01–2,34)	
Superior completo	1,00 (-)		1,00 (-)		1,00 (-)	1,00 (-)	1,00 (-)	
**Cor da pele**								
Branca	1,00 (-)		1,00 (-)		1,00 (-)	1,00 (-)	1,00 (-)	
Parda	1,12 (1,04–1,21)		1,12 (1,04–1,21)		1,66 (1,40–1,97)	1,40(1,18 – 1,66)	0,93 (0,60–1,42)	
Preta	1,18 (1,07–1,31)		1,16 (1,04–1,29)		1,70 (1,31–2,21)	1,44(1,12 – 1,85)	0,95 (0,55–1,64)	
**Local de moradia**								
Urbano	1,00 (-)	1,00 (-)	1,00 (-)	1,00 (-)	1,00 (-)	1,00 (-)	1,00 (-)	
Rural	0,82 (0,75–0,89)	0,82 (0,75–0,89)	0,82 (0,75–0,90)	0,83 (0,76–0,90)	0,86 (0,69–1,06)	0,77 (0,62–0,97)	1,09 (0,74–1,61)	
Socioeconômicas								
**Renda domiciliar**								
Até 1 SM	1,26 (1,12–1,42)		1,26 (1,11–1,42)		2,43 (1,86–3,16)		1,70 (1,04–2,77)	
Mais de 1 até 3 SM	1,05 (0,92–1,20)		1,05 (0,91–1,20)		1,45 (1,01–2,09)		1,33 (0,81–2,18)	
Acima de 3 SM	1,00 (-)		1,00 (-)		1,00 (-)		1,00 (-)	
**Estado Civil**								
Solteira	1,00 (-)	1,00 (-)	1,00 (-)	1,00 (-)	1,00 (-)	1,00 (-)	1,00 (-)	1,00 (-)
Casada	0,64 (0,59–0,70)	0,79 (0,72–0,86)	0,64 (0,59–0,70)	0,78 (0,71–0,86)	0,49 (0,37–0,64)	0,71 (0,54–0,94)	0,30 (0,19–0,47)	0,39 (0,21–0,70)
Viúva	0,43 (0,37–0,49)	0,69 (0,59–0,80)	0,43 (0,37–0,50)	0,69 (0,59–0,81)	0,27 (0,19–0,38)	0,64 (0,42–0,98)	0,16 (0,08–0,34)	0,35 (0,14–0,89)
Divorciada, desquitada ou separada judicialmente	0,98 (0,89–1,09)	1,15 (1,04–1,28)	0,99 (0,89–1,10)	1,16 (1,04–1,29)	0,79 (0,62–1,01)		0,82 (0,49–1,40)	
**Rede de apoio social**								
Nenhuma	1,57 (1,32–1,87)	1,36 (1,15–1,62)	1,56 (1,30–1,87)	1,35 (1,13–1,61)	2,21 (1,49–3,30)	1,57 (1,04–2,37)	1,63 (0,80–3,29)	
1 pessoa	1,46 (1,29–1,65)	1,28 (1,13–1,45)	1,46 (1,28–1,65)	1,28 (1,13–1,45)	1,96 (1,49–2,58)	1,44 (1,09–1,90)	2,05 (1,19–3,53)	
2 pessoas	1,26 (1,12–1,42)		1,28 (1,13–1,44)		1,59 (1,15–2,20)		1,30 (0,82–2,08)	
3 ou mais pessoas	1,00 (-)	1,00 (-)	1,00 (-)	1,00 (-)	1,00 (-)	1,00 (-)	1,00 (-)	
Saúde								
**Autoavaliação em saúde**								
Muito boa ou boa	1,00 (-)	1,00 (-)	1,00 (-)	1,00 (-)	1,00 (-)	1,00 (-)	1,00 (-)	1,00 (-)
Regular	1,49 (1,39–1,60)	1,51 (1,41–1,61)	1,49 (1,39–1,60)	1,50 (1,40–1,61)	1,51 (1,29–1,78)	1,46 (1,24–1,72)	1,54 (1,03–2,30)	1,50 (1,01–2,23)
Ruim ou muito ruim	1,69 (1,51–1,90)	1,65 (1,48–1,84)	1,74 (1,54–1,96)	1,68 (1,50–1,88)	2,29 (1,72–3,06)	2,01 (1,53–2 63)	2,18 (1,29–3,70)	1,96 (1,14–3,38)
**Consumo de álcool**								
Sim	1,67 (1,50–1,86)	1,38 (1,24–1,53)	1,66 (1,48–1,86)	1,37 (1,23–1,53)	2,65 (2,15–3,27)	1,91 (1,56–2,34)	2,99 (1,91–4,67)	2,14 (1,37–3,34)
Não	1,00 (-)	1,00 (-)	1,00 (-)	1,00 (-)	1,00 (-)	1,00 (-)	1,00 (-)	1,00 (-)
**Depressão**								
Sim	1,78 (1,65–1,92)	1,65 (1,54–1,78)	1,82 (1,69–1,97)	1,69 (1,57–1,82)	2,01 (1,68–2,40)	1,96 (1,65–2,32)	2,52 (1,73–3,68)	2,35 (1,65–3,35)
Não	1,00 (-)	1,00 (-)	1,00 (-)	1,00 (-)	1,00 (-)	1,00 (-)	1,00 (-)	1,00 (-)
**Infecções sexualmente transmissíveis**								
Sim	2,28 (1,82–2,86)	1,53 (1,22–1,91)	2,32 (1,84–2,92)	1,55 (1,23–1,95)	3,31 (2,08–5,26)	1,82 (1,10–3,02)	6,43 (3,41–12,12)	3,47 (1,89–6,37)
Não	1,00 (-)	1,00 (-)	1,00 (-)	1,00 (-)	1,00 (-)	1,00 (-)	1,00 (-)	1,00 (-)

*modelo multivariado

IC95%: intervalo de confiança de 95%; RPaj: razão de prevalência ajustada; SM: salário-mínimo.

## DISCUSSÃO

Aproximadamente uma em cada cinco mulheres brasileiras relatou algum tipo de violência nos 12 meses anteriores a entrevista, sendo o subtipo mais prevalente a psicológica. A maioria das mulheres relatou ter sofrido violência por um parceiro íntimo, na residência e terem consequências, principalmente de cunho psicológico. Dessas mulheres, aproximadamente um quinto procurou serviços de saúde, com até 6,50% delas necessitando internação por mais de 24 horas. A VCM associou-se a fatores demográficos, socioeconômicos e de saúde, mostrando sua relação macroestrutural com a sociedade.

A prevalência de VCM na PNS 2019 foi de 19,38%, similar a outros estudos^8 , 9^. Interessante notar que o Norte e o Nordeste contêm os estados que apresentaram as maiores prevalências de violência, mas também é nessas regiões que estão as menores prevalências do agravo. Essa discrepância pode ser reflexo da cultura machista dessas regiões, que normaliza a violência e dificulta seu reconhecimento pelas mulheres. Adicionalmente, há escassez de serviços nessas regiões, com menor acesso à informação e a redes de proteção, afetando diretamente a qualidade dos relatos^10^. Estudos anteriores^11 , 12^ apontaram essa variação subnacional, assim como mostraram que os maiores índices de subnotificação são encontrados nessas regiões^13^. Dessa forma, as políticas públicas precisam considerar as especificidades locais para melhorar o enfrentamento a violência, levando em conta os contextos e aspectos culturais de cada lugar.

Mesmo com números tão altos, é importante salientar que a prevalência da violência pode ser ainda maior. A violência ainda é um evento estigmatizado, sendo que muitas vezes as mulheres ocultam suas experiências, especialmente para estranhos para a realização de uma pesquisa^14^. A naturalização de relações abusivas e a dificuldade em identificar a violência em atos cotidianos são barreiras adicionais^14^. Nesse sentido, uma pesquisa realizada pelo DataSenado^15^ em 2023 revelou que, quando questionadas diretamente sobre terem sofrido algum tipo de violência nos últimos 12 meses, apenas 7,01% das mulheres brasileiras confirmaram tal vivência. No entanto, ao detalhar os atos violentos, com exemplos específicos, o percentual aumenta significativamente: 30,27% das mulheres relataram exposição a violência psicológica ou moral no período mencionado, enquanto 8,13% mencionaram violência física.

Deve-se destacar a alta prevalência da violência psicológica, sendo ela o subtipo com maior ocorrência isolada e concomitante com outros subtipos de violência. De fato, apenas 4,27% das mulheres relataram violências exclusivamente físicas e/ou sexuais. Dados semelhantes foram encontrados por estudos anteriores^10 , 14^. Esse achado mostra que a dominação da mulher muitas vezes acontece em patamar simbólico, sendo exercido por meio de ameaças e humilhação^16^. Além disso, desafia a visão tradicional que tende a hierarquizar os tipos de violências^14^. A violência psicológica não deve ser subestimada, nem vista como um prelúdio para formas “mais graves” de violência, como as físicas ou sexuais. Esse subtipo de violência é por si só uma grave violação da dignidade e saúde da mulher, acarretando impactos emocionais e mentais persistentes e profundos, uma realidade corroborada nesse estudo pelo elevado percentual de mulheres que relataram consequências psicológicas após a exposição a violência.

Esse estudo demonstrou que o principal agressor das mulheres foi um(a) parceiro(a) íntimo(a) e que o local mais comum de ocorrência da violência foi a residência, evidenciando a falácia da família protetora e do ambiente protegido do lar e reafirmando o papel do sistema patriarcal na raiz da VCM^2^. Dentro desse sistema, a violência surge como uma forma de manutenção da suposta supremacia dos homens, sexualizando as posições de poder e restringindo o acesso aos direitos por diferentes grupos sociais^17^. Portanto, é importante abordar o fenômeno da VCM em um contexto amplo, reconhecendo as estruturas de desigualdade que influenciam sua ocorrência e as dinâmicas sociais e culturais que perpetuam as iniquidades de gênero.

A maioria das mulheres relatou consequências decorrentes de violência, especialmente psicológicas, porém apenas um quinto delas procurou atendimento em saúde. Dentre essas mulheres, aquelas que sofreram violência sexual e física foram as que mais procuraram o atendimento em saúde, sendo essas últimas as com maior necessidade de internação. Esse padrão sugere que as mulheres brasileiras ainda veem o setor Saúde como local para resolução de consequências diretas da violência, como tratamento de lesões e profilaxia de ISTs, e não como porta de entrada para a Rede de Atendimento para mulheres que convivem com a violência^13^.

Esse estudo confirmou a correlação dos fatores demográficos e socioeconômicos com a VCM. Mulheres mais jovens, com menor escolaridade, pretas e pardas e com menor rede de apoio apresentaram maiores prevalências de violência. Esses elementos estão frequentemente associados a acesso restrito ao mercado de trabalho^18^, o que pode exacerbar a vulnerabilidade econômica e dependência financeira delas^19^. Embora a VCM não derive diretamente da pobreza, é necessário reconhecer o estresse enfrentado pela população economicamente vulnerável. Essas pessoas encontram piores oportunidades de trabalho, menor acesso a políticas públicas e desvalorização de seu poder aquisitivo, afetando negativamente o bem-estar individual e comunitário^20^. O estresse das iniquidades sociais vividas aumenta as chances do uso da violência para resolução de conflitos^21^, e as desvantagens econômicas das mulheres as impedem de se empoderar e desvencilhar de convívios violentos^17^. Por isso as Políticas Públicas eficazes no enfrentamento da VCM devem promover a autonomia feminina por meio do acesso amplo à educação, renda, moradia, entre outras, minimizando a exposição à violência.

As mulheres que residiam em zonas rurais apresentaram menores prevalências de exposição a violência. Alguns estudos atribuem as maiores prevalências de violência nas cidades ao processo de urbanização excludente do país, o que eleva as desigualdades sociais^22^. Porém é necessário se atentar ao silenciamento e invisibilidade das mulheres das zonas rurais. Uma revisão integrativa^23^ mostrou que essas mulheres têm alta exposição a violência, principalmente doméstica, e que esses atos acontecem de forma recorrente. No entanto questões culturais e religiosas impedem essas mulheres de romper o silêncio, sendo que as moradoras da zona rural podem demorar muitos anos para saírem de relacionamentos abusivos por ausência de conhecimento ou acesso a políticas públicas de empoderamento e proteção.

Também foi possível perceber a associação da VCM com fatores de saúde das mulheres brasileiras. A violência é um fator de risco para ansiedade e depressão, o que pode influenciar na percepção ruim da própria saúde^24^. Além disso, a violência contribui diretamente com sequelas físicas e ISTs, comprometendo a qualidade de vida das mulheres que convivem com ela^25^. Dados do *Global Burden of Disease* ^26^ (GBD) mostraram que, para as mulheres brasileiras em 2019, a violência interpessoal foi responsável por uma taxa de 44,65 anos vividos com incapacidades, além da violência por parceiro íntimo ter sido a causa atribuível de 7,11% das doenças depressivas e 7,99% de HIV/AIDS. Em relação ao consumo de álcool, sabe-se que mulheres alcoolistas apresentam maior vulnerabilidade, sujeitando-se à prostituição e violência para manutenção do vício.^27^ Além disso, o álcool tem papel no desequilíbrio das relações, podendo precipitar conflitos familiares^19^.

Os dados encontrados neste estudo evidenciam a forte associação da vivência de violência com fatores de vulnerabilidade da população. É importante destacar que o Brasil atravessou um período de austeridade, em que a limitação dos gastos sociais aprofundou as desigualdades presentes no país. Os desmontes das políticas públicas feitos entre 2016 e 2022 afetaram os progressos em relação à luta pela igualdade de gênero, com a diminuição do orçamento de equipamentos de amparo jurídico e assistencial às mulheres^28^. Além disso, medidas implantadas nesses anos facilitaram o acesso às armas, colocando em risco a vida das mulheres, especialmente aquelas em situação de violência^29^.

Este estudo analisou dados do ano anterior a pandemia por covid-19. As restrições sociais vinculadas às recomendações de saúde impuseram novas dinâmicas familiares e mulheres que conviviam com a violência ficaram ainda mais vulneráveis, uma vez que a interação com seus prováveis agressores aumentou, assim como o estresse econômico domiciliar^30^. Estudo anterior mostrou que, apesar de redução nas notificações, a VCM foi impactada pela pandemia, com maior número de denúncias telefônicas e um incremento nas solicitações de medidas protetivas^30^. Dessa forma, os dados da PNS 2019 servem como linha de base para novas análises sobre a prevalência da VCM durante e após a crise sanitária mundial.

Uma das limitações deste estudo é a restrição das perguntas do módulo de violência a mulheres acima de 18 anos, não permitindo ter uma visão desse agravo nas idades mais jovens. A PNS também não abrange populações de rua, asilos, quilombos e aldeias, e limita-se a avaliar apenas três subtipos de violência, omitindo a violência patrimonial, moral ou negligência, por exemplo. Essas exclusões levam a uma subestimação da prevalência, o que contribui para a invisibilidade do problema, especialmente entre mulheres em situações de maior vulnerabilidade. Além disso, o foco das características da violência é direcionado para o evento mais grave relatado nos últimos 12 meses, restringindo a compreensão da totalidade dos episódios de violência vivenciados por mulheres. Para tentar diminuir a subestimação da prevalência devido ao estigma da violência, procurou-se garantir a privacidade das mulheres no momento de resposta ao questionário, afastando-as de seu possível agressor, além de permitir resposta direta no dispositivo, diminuindo o possível constrangimento das respostas, mesmo com entrevistadores treinados para o contexto.

Em conclusão, a VCM tem alta prevalência no país, com aproximadamente um quinto da população feminina relatando ao menos um episódio de violência em um intervalo de 12 meses. Esse agravo está associado a fatores macroestruturais da sociedade, como racismo e sexismo, impactando diretamente na autonomia das mulheres, ao provocar sequelas de longo prazo e diminuir a qualidade de vida delas. Esses resultados evidenciam a relação das iniquidades em saúde e as vulnerabilidades sociais, o que intensifica a necessidade de articulação que envolva diversos setores da sociedade na criação de Políticas Públicas que assegurem a proteção da vida e dos direitos das mulheres.
